# SIRT7 promotes the proliferation and migration of anaplastic thyroid cancer cells by regulating the desuccinylation of KIF23

**DOI:** 10.1186/s12885-024-11965-9

**Published:** 2024-02-15

**Authors:** Yongkang Wu, Weijie Chen, Huilai Miao, Tuo Xu

**Affiliations:** 1 Department of Vascular and Thyroid Surgery, The Affiliated Hospital of Guangdong Medical University, No. 57, South Renmindadao, Xiashan District, Zhanjiang, Guangdong 524001 China; 2 Department of Hepatobiliary Surgery, The Second Affiliated Hospital of Guangdong Medical University, Zhanjiang, China

**Keywords:** Anaplastic thyroid cancer, KIF23, SIRT7, Succinylation, Proliferation, Migration

## Abstract

**Objective:**

This study was designed to investigate the regulatory effects of kinesin family member (KIF) 23 on anaplastic thyroid cancer (ATC) cell viability and migration and the underlying mechanism.

**Methods:**

Reverse transcription-quantitative polymerase chain reaction (RT-qPCR) was used to analyze the levels of KIF23 in ATC cells. Besides, the effects of KIF23 and sirtuin (SIRT) 7 on the viability and migration of ATC cells were detected using cell counting kit-8, transwell and wound healing assays. The interaction between SIRT7 and KIF23 was evaluated by co-immunoprecipitation (Co-IP) assay. The succinylation (succ) of KIF23 was analyzed by western blot.

**Results:**

The KIF23 expression was upregulated in ATC cells. Silencing of KIF23 suppressed the viability and migration of 8505C and BCPAP cells. The KIF23-succ level was decreased in ATC cells. SIRT7 interacted with KIF23 to inhibit the succinylation of KIF23 at K537 site in human embryonic kidney (HEK)-293T cells. Overexpression of SIRT7 enhanced the protein stability of KIF23 in HEK-293T cells. Besides, overexpression of KIF23 promoted the viability and migration of 8505C and BCPAP cells, which was partly blocked by silenced SIRT7.

**Conclusions:**

SIRT7 promoted the proliferation and migration of ATC cells by regulating the desuccinylation of KIF23.

**Supplementary Information:**

The online version contains supplementary material available at 10.1186/s12885-024-11965-9.

## Introduction

The incidence of thyroid cancer is increasing at an alarming rate, nearly tripling every decade [1]. Anaplastic thyroid cancer (ATC) is the most malignant thyroid cancer [1, 2]. ATC is undifferentiated, highly aggressive and mostly chemo-resistant. ATC patients often present with a neck mass causing dyspnea, dysphagia, dysphonia, and hoarseness [3]. The treatment options for ATC include surgical resection, external beam radiation therapy, and chemotherapy [4]. However, the prognosis of ATC patients is poor and nearly incurable with a median survival of only six months [5]. Thus, novel treatments for ATC are worth further exploration.

Kinesin family member (KIF) 23 is mainly located in the cytoplasm and nucleus and is widely involved in cell mitosis [6]. The abnormal function of KIF23 always results in the arrest of mitosis and the appearance of binuclear or multinucleated cells [7]. Studies have shown that the up-regulated expression of KIF23 is related to the occurrence and development of tumors [8, 9]. Recent research indicates that KIF23 plays an important role in the proliferation and migration of cancer cells [10–12]. Moreover, overexpression of KIF23 is associated with adverse clinical outcomes, which promotes the malignant behaviors of tumor cells through related signaling pathways [13]. However, the role of KIF23 in ATC has not been found.

Sirtuins (SIRTs) are nicotine adenine dinucleotide (+)-dependent histone deacetylases that regulate key signaling pathways in prokaryotes and eukaryotes. SIRTs are involved in many biological processes [14]. SIRT7 is a newly discovered protein of the SIRT family with relatively little research, which is localized to the nucleolar and controls the transcription of RNA polymerase I [15]. A previous study indicates that SIRT7 is increased in tumor tissues compared with normal tissues [16]. SIRT7 promotes the proliferation and invasion of tumor cells by activating related signaling pathways [17]. In addition, a study indicates that SIRT7 is overexpressed in human thyroid cancer cells and tissues [18]. Whereas, the effects of SIRT7 and the relationship between SIRT7 and KIF23 in ATC have not been explored.

Succinylation is a newly discovered post-translational modification of proteins, which is an important regulatory mechanism affecting protein function [19]. Studies have shown that succinylation alters the processes of enzymes and metabolic pathways and is linked to diseases of the liver, heart, lung, and other organs [20, 21]. Research shows that succinylation affects thyroid hormone synthesis, inhibits the migration and promotes apoptosis of thyroid cancer cells [22]. Nevertheless, the specific regulatory mechanism of succinylation in ATC remains unclear.

Given this background, this study aimed to explore the effects of KIF23 on ATC cell viability and migration and the underlying mechanism, which might provide a potential therapeutic intervention strategy for ATC.

## Methods and materials

### Antibodies

The antibodies used in this study were listed as follows: KIF23 (Santa Cruz Biotechnology, sc-390113), SIRT7 (Santa Cruz Biotechnology, sc-365344), succinyl lysine (PTM Biolabs, Hangzhou, China; PTM-419), glyceraldehyde 3-phosphate dehydrogenase (GAPDH) (Thermo Fisher, MA5-15738-D680), and IgG (Abcam, ab6715). Normal goat anti-rabbit (35,552) and goat anti-mouse (35,502) IgG were purchased from Thermo Fisher.

### Bioinformatic analysis

The GSE85457 dataset was acquired from Gene Expression Omnibus (GEO, https://www.ncbi.nlm.nih.gov/geo/). R language was used to analyze the data, and the differentially expressed genes were defined as *p* < 0.05 and |log2(fold change)|>1. The starbase database (https://rnasysu.com/encori/panCancer.php) was used to analyze the expression of KIF23 in 510 cancer and 58 normal samples in thyroid carcinoma (THCA). The LinkedOmics database (https://www.linkedomics.org/login.php) showed genes positively and negatively related to KIF23. The SuccinSite and GPSuc databases (http://systbio.cau.edu.cn/SuccinSite/; http://kurata14.bio.kyutech.ac.jp/GPSuc/index.php) were used to screen succinylation sites of KIF23.

### Cell culture

ATC cells (TPC1, 8305C, 8505C, and BCPAP), thyroid epithelial cell-derived cell line (HTori3), and human embryonic kidney (HEK)-293T cells were purchased from the Cell Bank of the Chinese Academy of Sciences (Shanghai, China). ATC cells and HTori3 cells were cultured in a complete medium (RPMI-1640; Invitrogen, Carlsbad, CA, USA) containing 10% heat-inactivated fetal bovine serum (FBS, Invitrogen), 100 U/mL penicillin, and 100 µg/mL streptomycin (Invitrogen). HEK-293T cells were maintained in the DMEM (Thermo Fisher Scientific, Waltham, MA, USA) containing 10% FBS and 1% penicillin/streptomycin. All cells were incubated in a humidified incubator at 37 °C with 5% CO_2_.

### Reverse transcription-quantitative polymerase chain reaction (RT-qPCR)

Total RNA from cells was extracted by TRIzol reagent (Invitrogen). Then, RNA was reverse transcribed into cDNA using the PrimeScript RT kit (Takara, Dalian, China). The qPCR amplification experiment was performed using the Taq Pro Universal SYBR qPCR Master Mix kit (Vazyme, Nanjing, China) with the reaction conditions: a cycle of 95 °C for 30 s, 40 cycles of 95 °C for 10 s and 60 °C for 30 s, and a cycle of 95 °C for 15 s, 60 °C for 60 s and 95 °C for 15 s. The gene expression was calculated by the 2^−ΔΔCT^ method. Primers used in this study were synthesized by Sangon Biotechnology Co., LTD (Shanghai, China) and listed in Table 1.

**Table 1:** List of siRNAs and qPCR primes used in this paper

Gene	Primer	Sequence(5’-3’)
si-KIF23#1	Forward	GACUAUAUCUAGAUCAUGUCU
	Reverse	ACAUGAUCUAGAUAUAGUCUU
si-KIF23#2	Forward	GAAGUGAUCAAUAAUACAACU
	Reverse	UUGUAUUAUUGAUCACUUCUA
si-NC	Forward	UUCUCCGAACGUGUCACGUTTU
	Reverse	ACGUGACACGUUCGGAGAATT
si-SIRT7	Forward	GCAGCCUCUAUCCCAGAUUTT
	Reverse	AAUCUGGGAUAGAGGCUGCTT
GAPDH	Forward	CCACCCATGGCAAATTCCATGGCA
	Reverse	TCTAGACGGCAGGTCAGGTCCACC
KIF23	Forward	CTGACCCAGAGCAAAGCTTTC
	Reverse	GTTCTAAAGTGCATTCTTGCAGC

*KIF23* Kinesin family member 23, *GAPDH* Glyceraldehyde-3-phosphate dehydrogenase, *SIRT* Sirtuin, *siRNA* Small interfering RNA, *qPCR* Quantitative polymerase chain reaction

### Cell transfection

KIF23 small interfering RNAs (si-KIF23#1/2), SIRT7 siRNA (si-SIRT7), siRNA negative control (si-NC), pcDNA3.1-KIF23 overexpression vector, and the empty vector (pcDNA3.1) used in this study were purchased from GenePharma (Shanghai, China). The HEK-293T, 8505C, and BCPAP cells (5 × 10^5^ cells/well) were inoculated in a 6-well plate (Corning, NY, USA). After the cell confluence reached 80%, transfection was performed using Lipofectamine 2000 (Invitrogen) according to the manufacturer’s instructions. The cells were transfected for 48 h, and the transfection efficiency was detected by RT-qPCR. The primers used in cell transfection were synthesized by Sangon and listed in Table 1.

Flag-KIF23-K437S and Flag-KIF23-K537S were designed by Genscript Biotechnology Co., LTD (Nanjing, China). Briefly, serine mutations were introduced at K437 (K437S) and K537 (K537S) sites of KIF23. Then, the Flag-KIF23-WT, Flag-KIF23-K437S, and Flag-KIF23-K537S plasmids were transfected into HEK-293T cells for 24 h.

### Cell counting kit-8 (CCK-8) assay

Cell viability was detected by the CCK-8 kit (Dojindo, Japan). The cells were seeded into a 96-well plate at the density of 1 × 10^3^ cells/well. Three replicate wells were set up. The cells were maintained in the incubator for 24 h, and 10 µL of CCK-8 solution was added to each well to incubate with cells for 2 h. The absorbance was assessed at 450 nm using a microplate reader (Synergy HT, Bio-Tek, USA).

### Transwell migration assay

Cells with the serum-free medium were seeded to the upper transwell chamber (Costar, USA). The bottom of the chamber was supplied with RPMI-1640 medium containing 10% FBS. After 24 h, the migrated cells were washed twice with sterile phosphate buffer saline (PBS, Solarbio), fixed in 4% paraformaldehyde (Solarbio, China) for 30 min, and then stained with 0.1% crystal violet (Solarbio) for 20 min. The migrated cells were imaged with a light microscope (Olympus, Japan), and the stained cell number was calculated.

### Wound healing assay

ATC cells were seeded onto the 6-well plates. When cell confluence reached about 95%, a 200 µL pipette tip was used to create the scratches on the cell monolayer, and cell images were taken at 0 and 24 h, under an optical microscope (Olympus, Japan). The wound healing area was measured by the Image-Pro Plus 6.0 software. The wound healing assay was conducted three times with three biological repetitions each time.

### Co-immunoprecipitation (Co-IP) and IP assay

Co-IP was used to screen proteins related to the succinylation of KIF23 and to verify the interaction between KIF23 and SIRT7 in HEK-293T cells. Cells were lysed on ice in RIPA buffer (Beyotime Biotechnology, Shanghai, China) containing protease inhibitors for 30 min. The supernatant was collected and 10 µL of it was taken as the input group. After that, KIF23, SIRT7, or lgG antibody (2 µg) was added into the supernatant for incubation overnight at 4 °C. The protein A agarose beads (Thermo Fisher) were washed with appropriate lysis buffer (Beyotime) three times. The pre-treated 10 µL protein A agarose beads were then added to the cell lysate and antibody complex, and slowly shook at 4 °C for 2 h to make the antibody conjugated with the protein A agarose beads. After the immunoprecipitation reaction, the complex was centrifuged at 4 °C, 3,000 rpm for 3 min. Next, the supernatant was discarded and the agarose beads were washed with 1 mL of lysis buffer three times. Finally, 15 µL of 2 × SDS loading buffer (Beyotime) was added and boiled for 5 min. The precipitated protein was then analyzed using western blot assay.

### Western blot

The cells were harvested and the lysis buffer was used to extract the protein. After the protein concentration was determined by the BCA method (Thermo Fisher), 50 µg of protein was separated by 10% SDS-PAGE, and then transferred to the PVDF membrane (Thermo Fisher). The membrane was blocked in 5% skim-milk for 1 h, then incubated with the primary antibodies at 4 °C overnight. The membrane was washed three times with Tris-buffered saline Tween (TBST, Thermo Fisher), and the secondary antibody was incubated with the membrane for 1 h at room temperature. Finally, an enhanced chemiluminescence solution (Pierce; Thermo Fisher) was used for protein signal detection.

### Protein stability assessment

KIF23 protein stability assessment was performed to verify the stability of KIF23 when SIRT7 was overexpressed in HEK-293T cells. HEK-293T cells were treated with cycloheximide (CHX, 100 µg/mL, Abcam, USA), a protein translation inhibitor [23], for 0, 6, 12, and 24 h. The protein level of KIF23 was measured by western blot.

### RNA stability assessment

RNA stability assessment was performed to verify the stability of KIF23 after SIR7 overexpression in HEK-293T cells. HEK-293T cells were treated with actinomycin D (ACD, 0.5 µg/mL, Sigma), then existing KIF23 expression was detected at different time point (0, 6, 12, and 24 h) using qPCR.

### Immunofluorescence (IF) staining

IF assay was performed to observe the distribution of SIRT7 and KIF23 in HEK-293T cells. The cells growing on the glass slide were soaked in PBS for 3 times, and fixed with 4% paraformaldehyde for 15 min. Next, the sections were rinsed three times with PBS, and incubated with SIRT7 and KIF23 antibodies overnight at 4 °C overnight, followed by incubation with the secondary antibody for 1 h at room temperature away from light. Finally, the sections were mounted with Antifade Mounting solution containing 10 mg/mL 4',6-diamidino-2-phenylindole (DAPI, Beyotime, China). Representative visual fields were acquired using a Leica DM5000 B microscope (Leica Microsystems, Wetzlar, Germany).

### Statistical analysis

The SPSS 21.0 software was used to analyze data. Data are expressed as mean ± standard deviation (SD). Student’s t-test was used for comparison between the two groups. One-way analysis of variance (ANOVA) was used for comparison among groups. Statistical analyses were performed using the GraphPad Prism software (v8.0.1, GraphPad Software Inc., San Diego, CA, USA). *p* < 0.05 indicates that the difference is statistically significant.

## Results

### High expression of KIF23 in ATC cells

**Fig. 1:**
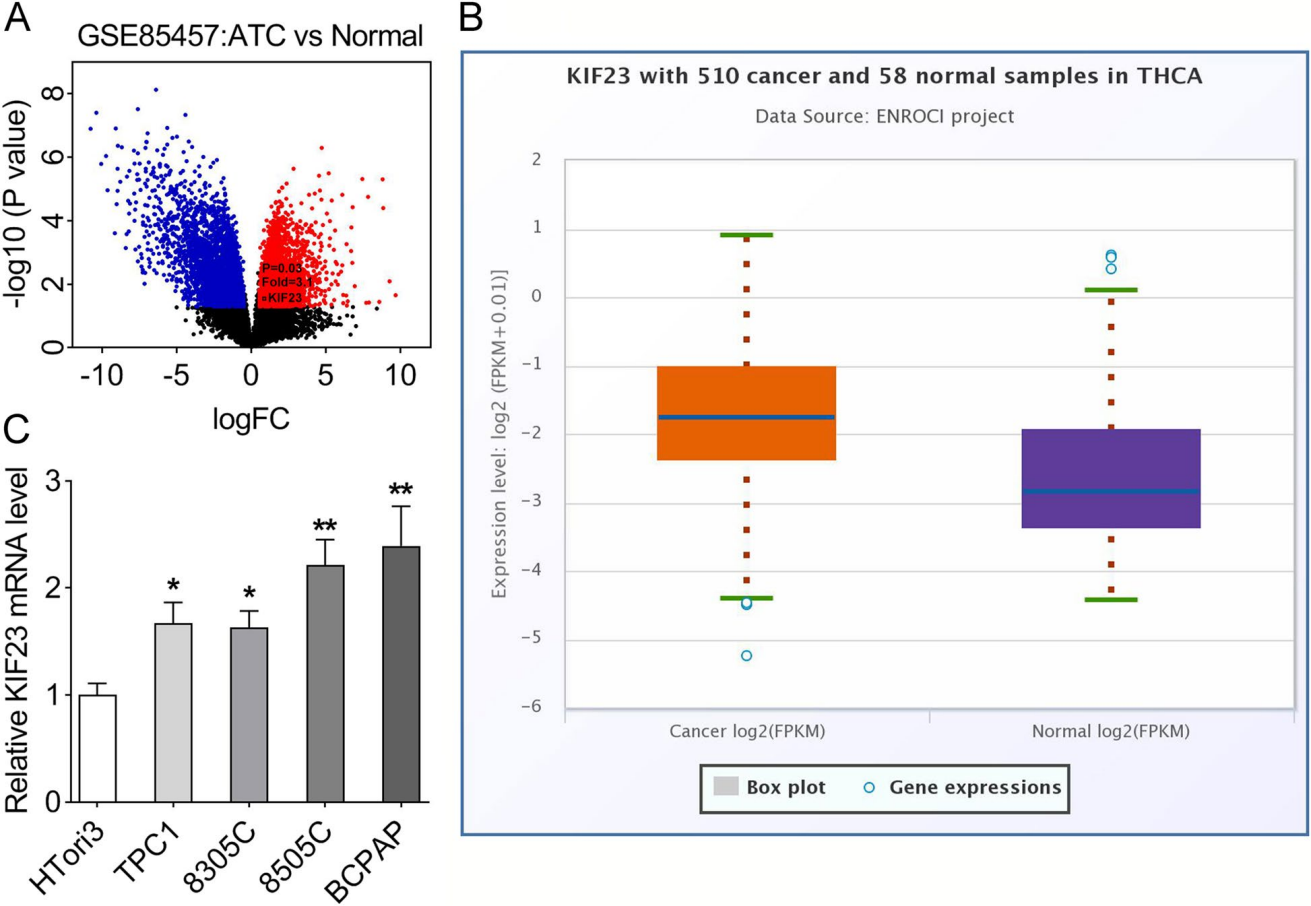
KIF23 was highly expressed in ATC cells. **A** The volcanic map showed differentially expressed genes in ATC vs. normal samples. Red represents up-regulated genes and blue represents down-regulated genes; **B** The expression of KIF23 in 510 cancer and 58 normal samples in THCA was analyzed by the starbase database; **C** RT-qPCR assay was performed to analyze the expression level of KIF23 in ATC cells (TPC1, 8305C, 8505C, BCPAP) and thyroid epithelial cell-derived cell line (HTori3). (*p < 0.05; **p < 0.01). KIF23, kinesin family member 23; ATC, anaplastic thyroid cancer; RT-qPCR, reverse transcription-quantitative polymerase chain reaction; THCA, thyroid carcinoma

We used the volcanic map to show the differentially expressed genes in ATC and normal samples (Fig. 1A). In addition, THCA database showed that KIF23 was upregulated in cancer samples compared with the normal samples in thyroid carcinoma (Fig. 1B). Moreover, the KIF23 expression was elevated in ATC cells (TPC1, 8305C, 8505C, BCPAP), compared with the thyroid epithelial cell-derived cell line (HTori3) (Fig. 1C). The 8505C and BCPAP cells were chosen for the following experiments.

### Down-regulation of KIF23 inhibited cell viability and migration of ATC cells

**Fig. 2:**
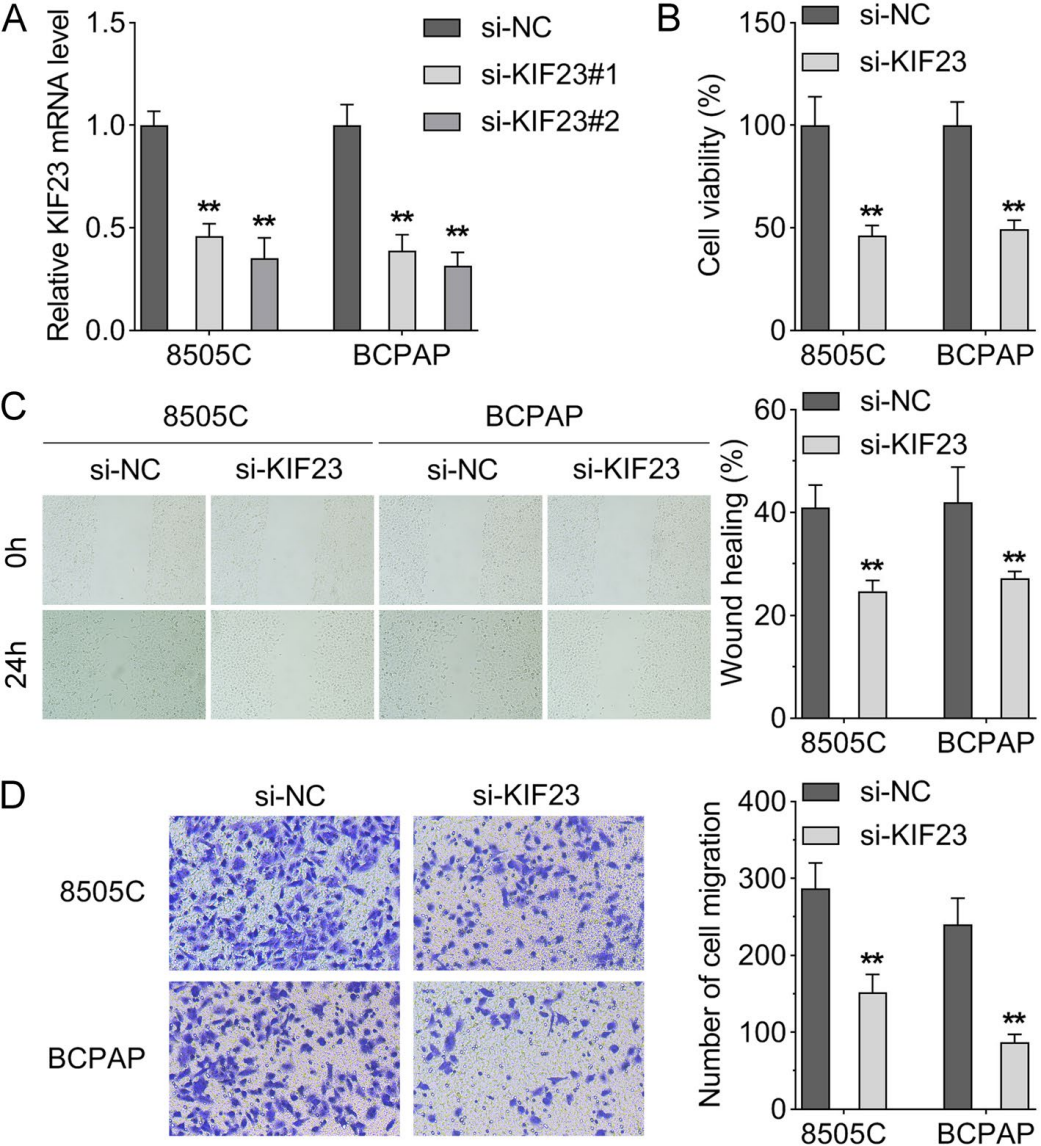
The silence of KIF23 inhibited the viability and migration of 8505C and BCPAP cells. **A** The transfection efficiency of si-KIF23#1/2 in 8505C and BCPAP cells was affirmed by RT-qPCR; **B** CCK-8 assay was performed to assess the viability of 8505C and BCPAP cells when KIF23 was knocked down; **C** Wound healing assay was used to evaluate the migration of 8505C and BCPAP cells after silencing KIF23; **D** Transwell migration assay was used to detect 8505C and BCPAP cell migration after silencing of KIF23. (**p < 0.01). KIF23, kinesin family member 23; RT-qPCR, reverse transcription-quantitative polymerase chain reaction; siRNA, small interfering RNA; CCK-8, cell counting kit-8

To further explore the biological functions of KIF23 in ATC progression, we transfected si-KIF23#1/2 into 8505C and BCPAP cells. The results of transfection efficiency showed that the KIF23 expression was downregulated after si-KIF23#1/2 transfection (Fig. 2A). CCK-8 assay results indicated that the silence of KIF23 distinctly decreased the cell viability of 8505C and BCPAP cells (Fig. 2B). Additionally, the number of migrated cells and wound healing area were also decreased by KIF23 knockdown (Fig. 2C and D).

### Silencing of SIRT7 increased KIF23-succ level in HEK-293T cells

**Fig. 3:**
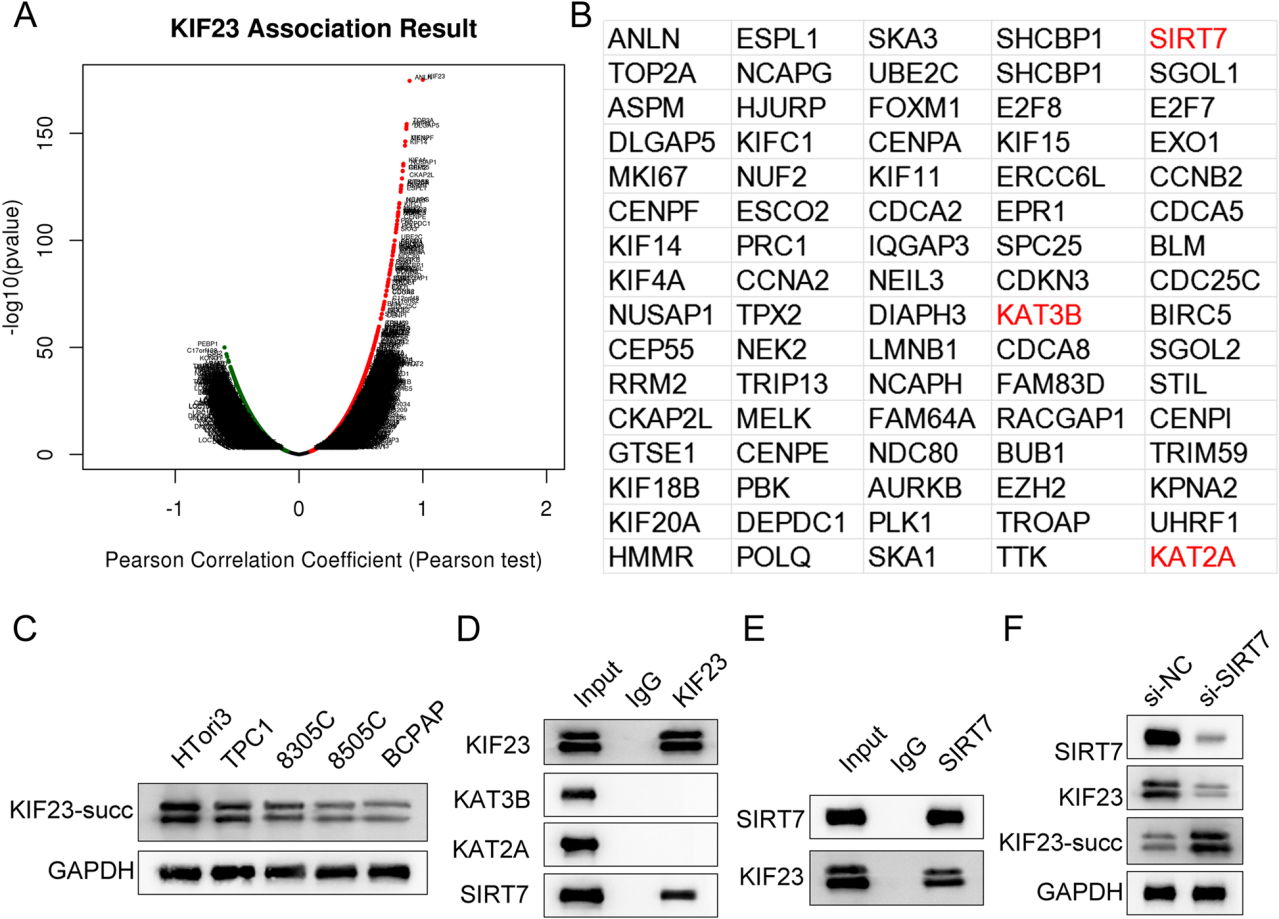
Silencing of SIRT7 increased KIF23-succ level in HEK-293T cells. **A** Genes positively and negatively related to KIF23 were obtained using the Linkedomics database; **B** The 80 genes positively related to the expression of KIF23. Red represents the genes associated with succinylation; **C** The levels of KIF23-succ in HTori3 and ATC cells (TPC1, 8305C, 8505C, BCPAP) were detected by Western blot; **D** Co-IP assay was used to screen the proteins associated with succinylation of KIF23 in HEK-293T cells; **E** Co-IP assay was used to detect the interaction between KIF23 and SIRT7 in HEK-293T cells; **F** Western blot was used to detect the levels of SIRT7, KIF23, and KIF23-succ after downregulation of SIRT7 in HEK-293T cells. KIF23, kinesin family member 23; ATC, anaplastic thyroid cancer; Co-IP, Co-immunoprecipitation; HEK, human embryonic kidney; SIRT, sirtuin; succ, succinylation

The results from the LinkedOmics database showed the genes positively and negatively correlated with KIF23 (Fig. 3A). We screened 80 genes positively correlated with KIF23 for analysis and found that there were representative genes [lysine acetyltransferase (KAT) 2A, KAT3B, and SIRT7] related to succinylation modification (Fig. 3B). Therefore, we speculated that KIF23 was succinylated in ATC and examined the level of succinylation of KIF23. The results indicated that the succinylation level of KIF23 in ATC cells (TPC1, 8305C, 8505C, BCPAP) was lower than that in HTori3 cells (Fig. 3C). Moreover, Co-IP assay was used to screen for protein associated with succinylation of KIF23. The results demonstrated that KIF23 interacted with SIRT7, but could not interact with KAT3B and KAT2A in HEK-293T cells (Fig. 3D and E). Besides, silencing of SIRT7 decreased the protein levels of KIF23 and SIRT7, and increased the levels of KIF23-succ in HEK-293T cells (Fig. 3F). These results demonstrated that KIF23 was desuccinylated by SIRT7 in ATC.

### KIF23 was desuccinylated at K537 site

**Fig. 4:**
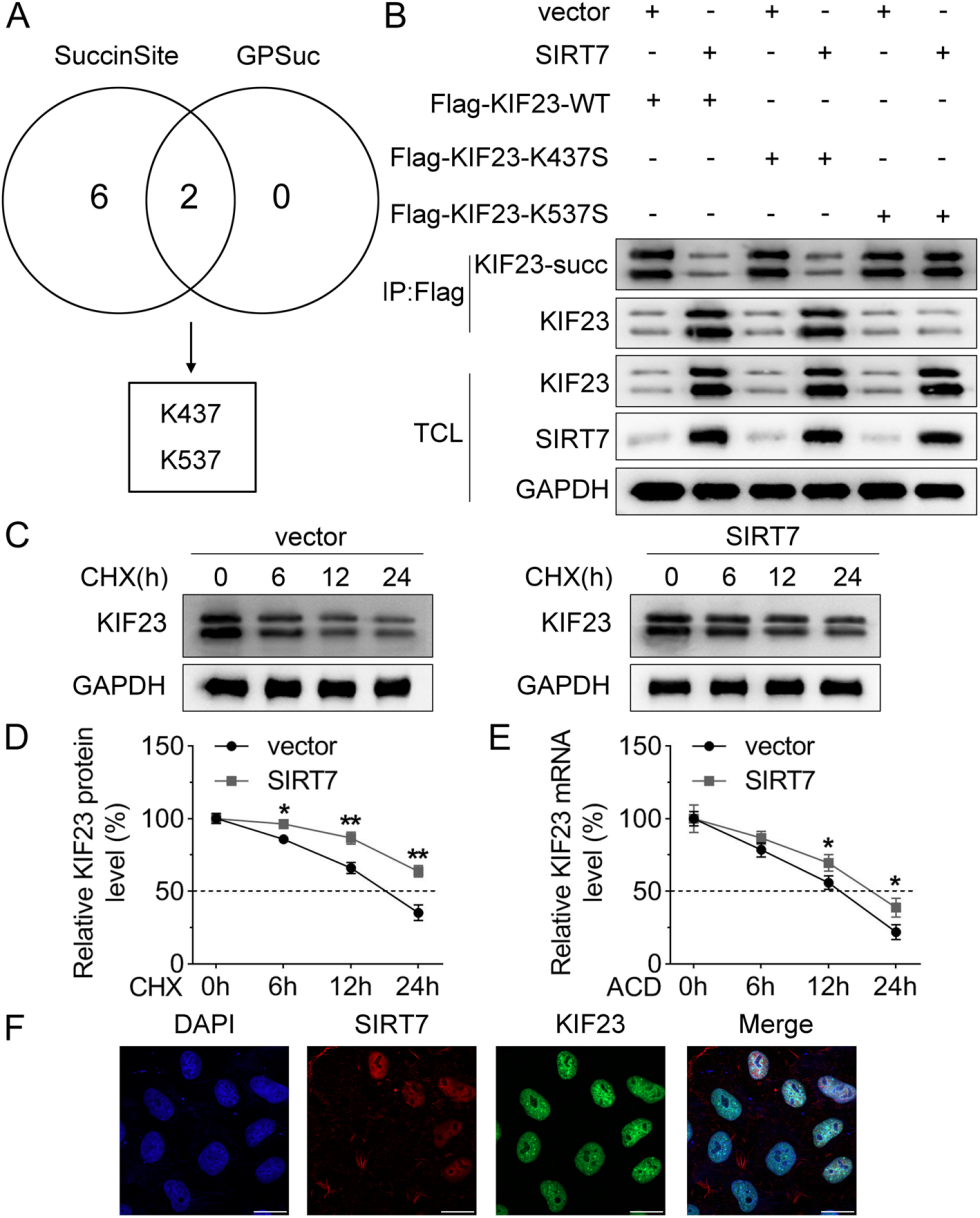
KIF23 was desuccinylated at K537 site. **A** The succinylation sites for KIF23 were predicted using the SuccinSite and GPSuc databases; **B** IP assay was used to analyze the succinylation sites of KIF23; **C** The HEK-293T cells transfected with an empty vector and SIRT7 overexpression vector were treated with CHX, then the protein expression of KIF23 was assayed by western blot at the different time points (0, 6, 12, and 24 h); **D** The quantitative analysis of KIF23 protein stability; **E** RNA stability assay was used to detect the existing KIF23 expression when actinomycin D treated at different time points (0, 6, 12, and 24 h); **F** IF ataining was performed to analyze the protein distribution of KIF23 and SIRT7 in HEK-293T cells. DAPI was used to stain the nuclei. KIF23, kinesin family member 23; IP, immunoprecipitation; HEK, human embryonic kidney; SIRT, sirtuin; CHX, cycloheximide; succ, succinylation; IF, immunofluorescence; DAPI, 4’,6-diamidino-2-phenylindole

Furthermore, we used the SuccinSite and GPSuc databases to predict KIF23 succinylation sites, and the results showed two possible KIF23 succinylation sites, K437 and K537 (Fig. 4A). Then, serine mutations were introduced at K437 and K537 sites of KIF23. IP results showed that overexpression of SIRT7 decreased the KIF23-succ level and increased the KIF23 protein level. SIRT7 downregulated the KIF23-succ protein level and increased the KIF23 protein level when co-transfected with Flag-KIF23-K437S rather than Flag-KIF23-K537S, suggesting that KIF23 was desuccinylated at K537 site (Fig. 4B). Protein and stability assay revealed that overexpression of SIRT7 enhanced the protein stability of KIF23 in HEK-293T cells (Fig. 4C and D). Moreover, the mRNA stability results indicated that after 12 h of ACD treatment, overexpression of SIRT7 increased the mRNA stability of KIF23 (Fig. 4E). IF staining was used to verify the relationship between KIF23 and SIRT7. The results suggested that SIRT7 co-located with KIF23 in HEK-293T cells (Fig. 4F).

### Silencing of SIRT7 reversed cell viability and migration of 8505C and BCPAP cells induced by KIF23

**Fig. 5:**
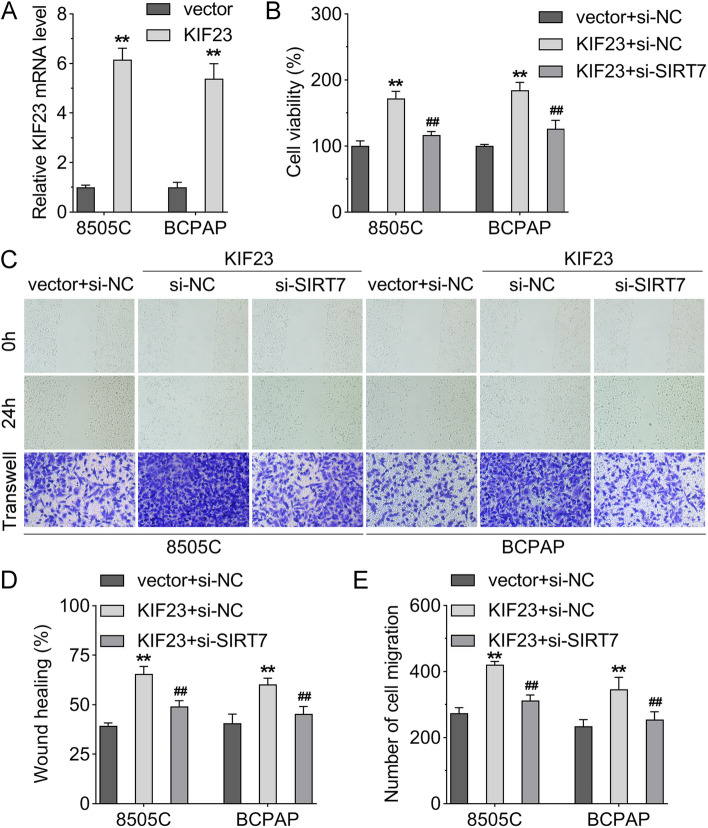
Silencing of SIRT7 reversed cell viability and migration of 8505C and BCPAP cells induced by KIF23. **A** Overexpression KIF23 in 8505C and BCPAP cells; **B** CCK-8 assay was performed to assess the cell viability of 8505C and BCPAP cells transfected with si-RNA negative control (si-NC), SIRT7 knockdown plasmid (si-SIRT7) and KIF23 overexpression vector; **C** Wound healing and transwell migration assays were used to evaluate cell migration of 8505C and BCPAP cells in each group; **D** The wound healing area and **E** the number of migrated cells of each group. (**p < 0.01 vs. vector + si-NC group; ^##^*p* < 0.01 vs. KIF23 + si-NC group). KIF23, kinesin family member 23; HEK, human embryonic kidney; SIRT, sirtuin; siRNA, small interfering RNA; CCK-8, cell counting kit-8

KIF23 overexpression plasmid was transfected into 8505C and BCPAP cells, and its expression was increased (Fig. 5A). Moreover, overexpressing KIF23 enhanced the cell viability in 8505C and BCPAP cells, while silencing of SIRT7 restored the result (Fig. 5B). Besides, the cell migration of 8505C and BCPAP cells was promoted when KIF23 was overexpressed, which was reversed after SIRT7 silencing (Fig. 5C-E).

## Discussion

KIF23 is proven to be involved in the progression of various tumors [23]. In our study, we found that the expression of KIF23 was increased in ATC cells compared with that in normal cells. A similar study shows that KIF23 is enriched in cell cycle programs in patients with thyroid-associated ophthalmopathy [24]. Besides, KIF23 is significantly up-regulated and associated with poor prognosis in different kinds of cancers [12, 25]. Moreover, KIF23 is correlated with multiple aspects of tumors including prognosis, clinical grade, stage, immune subtype, immune microenvironment, etc [26]. The cell viability and migration of 8505C and BCPAP cells in our study were all suppressed after KIF23 silencing, suggesting that KIF23 may promote the progression of ATC. Similarly, KIF23 promotes the proliferation, migration, and invasion of various tumors in vitro and in vivo, playing a tumorigenic role in cancer progression [25, 27, 28]. Subsequently, we explored the potential mechanism by which KIF23 promoted the progression of ATC.

Protein succinylation is mainly regulated by succinyl donors including succinyltransferases and desuccinylases. This process mainly occurs in the cytoplasm and nucleus [19]. Increasing evidence demonstrates that the modulators of succinylation promote or inhibit a variety of cancers by modulating the level of succinylation at substrate targets [29, 30]. A study has shown that abnormal thyroid-stimulating hormone secretion caused by thyroglobulin mutation may induce thyroid cancer, which may be related to succinylation [19]. We found that the KIF23-succ level was decreased in ATC cells. Thus, we speculated that the effect of KIF23 on ATC cell viability and migration might be associated with its desuccinylation. Previous studies have found that phosphorylation and DNA methylation modifications in KIF23, are linked to several diseases [31, 32]. However, the regulator of the succinylation of KIF23 has not been found. In the present study, SIRT7 interacted with KIF23 in HEK-293T cells. In addition, silencing of SIRT7 decreased SIRT7 and KIF23 protein levels but enhanced that of KIF23-succ, which was consistent with a recent study indicating that SIRT7 exerts inhibitory effects on KIF23 succinylation [33]. Moreover, a previous study shows that overexpressing SIRT7 promotes the proliferation and invasion of tumor tissues through its-related signaling pathway, which is similar with our results [17]. Whether there are other post-translational modifications of KIF23 in ATC needs further studied. Interestingly, our results found that KIF23 was desuccinylated at K537 site, which has never been reported in previous studies. This could provide a reference for further study to explore the specific mechanism of KIF23 in ATC or other cancers. However, due to experimental conditions, we were unable to use mass spectrometry to strengthen the results that SIRT7 desuccinylated K537 site of KIF23, which will be remedied in the future.

In summary, this study indicated that SIRT7 promoted the proliferation and migration of ATC cells by regulating the desuccinylation of KIF23, which might provide new ideas for the clinical treatment of ATC. However, there are still some limitations in this study, for example, the results of this study only represent experiments in vitro, and the results in vivo will be further analyzed in future studies.

### Supplementary Information

**Supplementary material 1.**

## Data Availability

The datasets used and/or analyzed during the current study are available from the corresponding author on reasonable request.
