# Supplementary material for: SIRT7 promotes the proliferation and migration of anaplastic thyroid cancer cells by regulating the desuccinylation of KIF23
Source: BMC Cancer. 2024 Feb 15;24:210. doi: 10.1186/s12885-024-11965-9 (PMC10870498; doi:10.1186/s12885-024-11965-9)
Supplement: Supplementary file 1 — Supplementary material 1. [file 12885_2024_11965_MOESM1_ESM.docx]

Figure 3-C

| 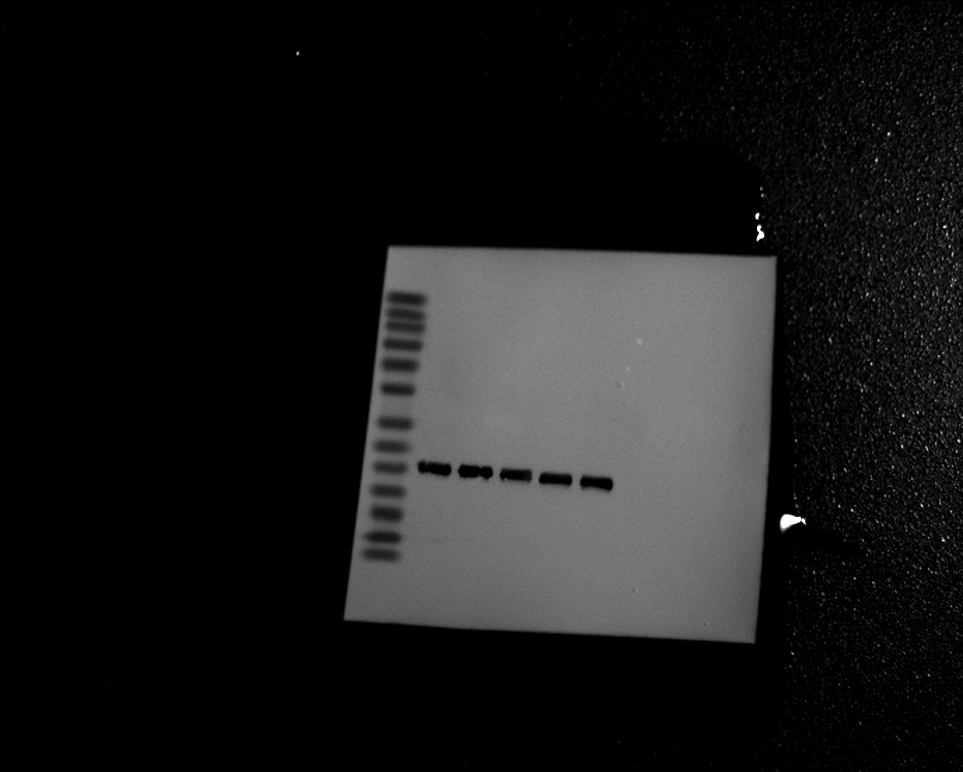 | 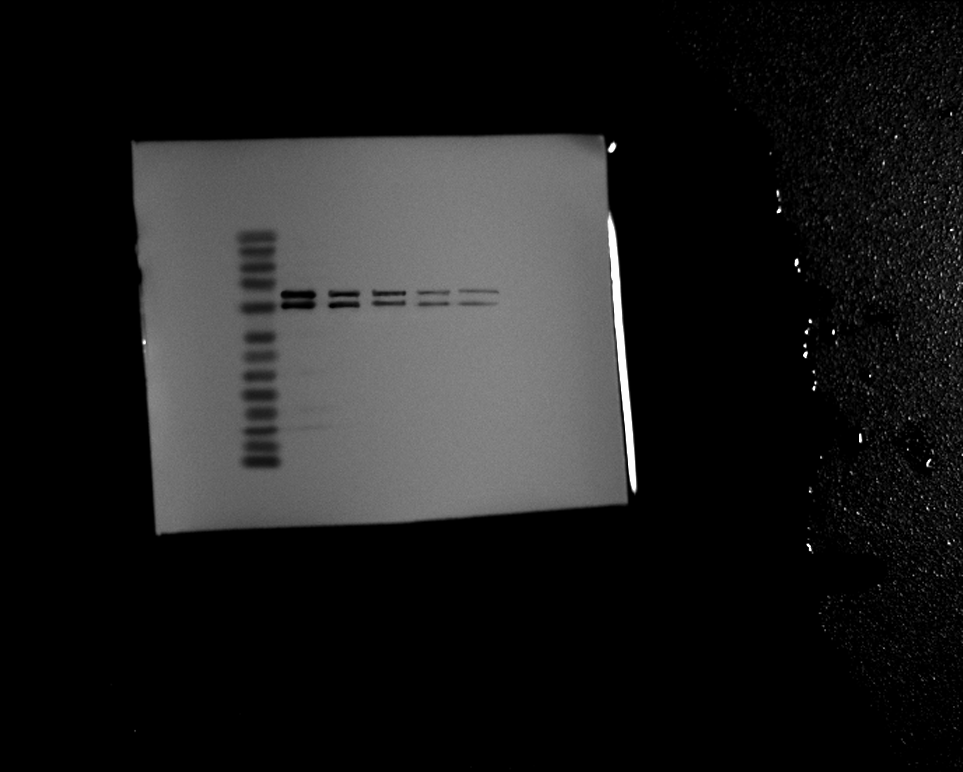 |
| --- | --- |
| GAPDH | KIF23-succ |

Figure 3-D

| 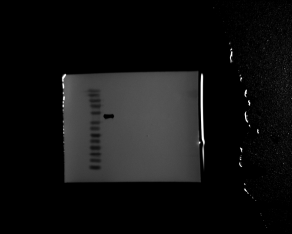 | 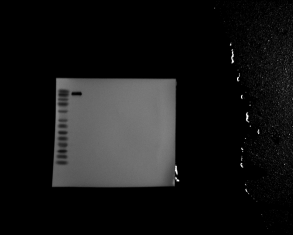 | 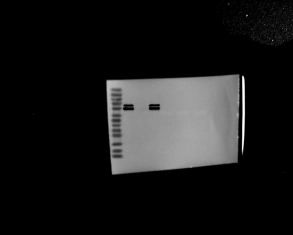 | 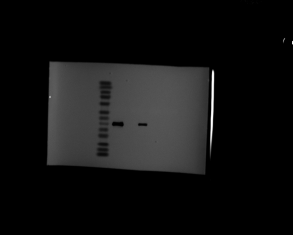 |
| --- | --- | --- | --- |
| KAT2A | KAT3B | KIF23 | SIRT7 |

Figure 3-E

| 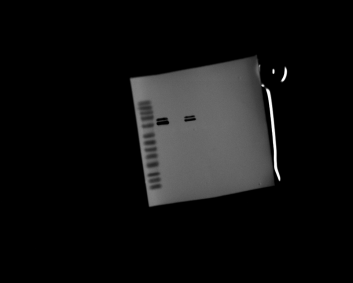 | 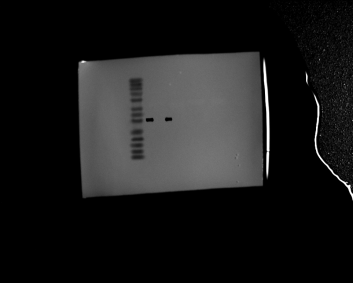 |
| --- | --- |
| KIF23 | SIRT7 |

Figure 3-F

| 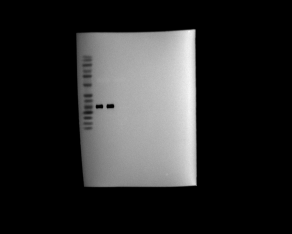 | 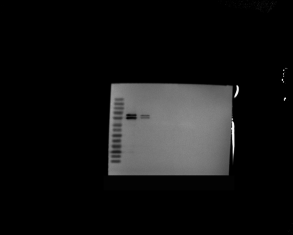 | 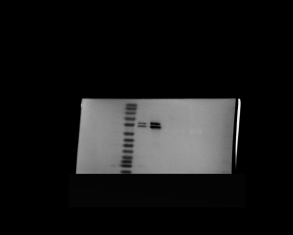 | 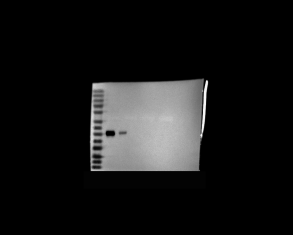 |
| --- | --- | --- | --- |
| GAPDH | KIF23 | KIF23-succ | SIRT7 |

Figure 4-B

| 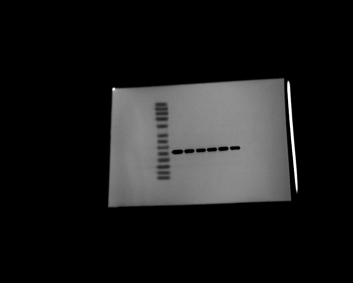 | 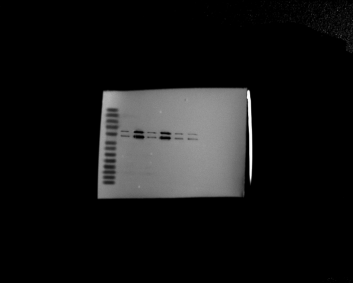 | 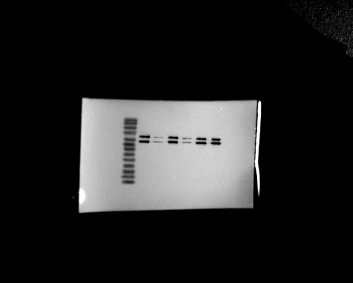 |
| --- | --- | --- |
| GAPDH | KIF23 | KIF23-succ |
| 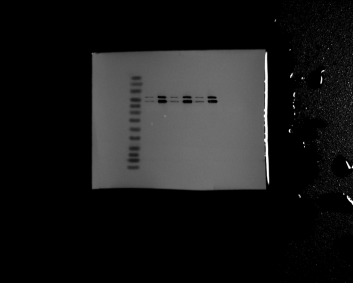 | 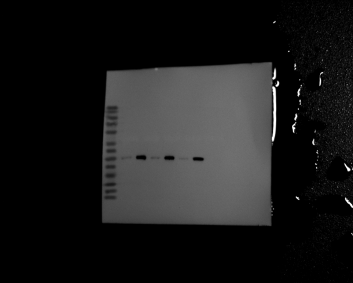 |  |
| KIF23-TCL | SIRT7 |  |

Figure 4-C

| 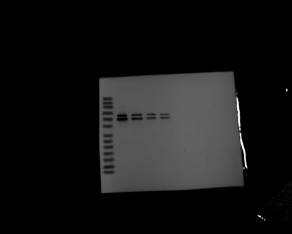 | 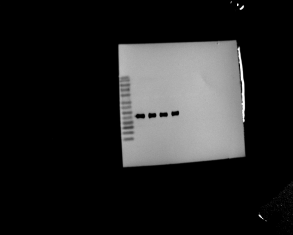 | 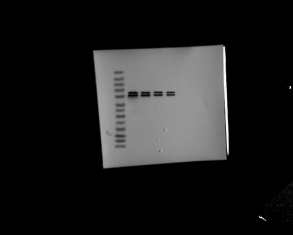 | 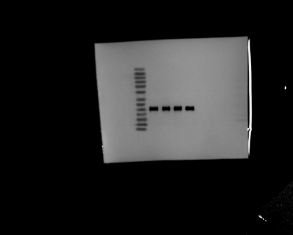 |
| --- | --- | --- | --- |
| 1.KIF23 (1) | 2.GAPDH (1) | 3.KIF23 (2) | 4.GAPDH (2) |
